# An Unusual Case of Acute Appendicitis due to Metastatic Prostatic Adenocarcinoma

**DOI:** 10.1155/2020/9430452

**Published:** 2020-08-18

**Authors:** Numbereye Numbere, Andrew Dunn, Aaron R. Huber

**Affiliations:** ^1^Department of Pathology and Laboratory Medicine, University of Rochester Medical Center, Rochester, New York, USA; ^2^Department of Pathology, Hattiesburg Clinic, Hattiesburg, Mississippi, USA

## Abstract

Acute appendicitis is a common surgical emergency in older adults. In the elderly, like in younger cohorts, acute appendicitis most commonly arises without neoplastic underpinnings. However, the occurrence of acute appendicitis in a patient with a concurrent abdominopelvic malignancy should trigger suspicion for the possibility of a metastatic appendiceal neoplasm. We present the case of a 66-year-old man with a background of a biochemically recurrent prostatic adenocarcinoma who presented to the emergency department with acute appendicitis. Histopathologic examination of the resected appendix revealed an unexpected metastatic spread from his prostatic adenocarcinoma.

## 1. Introduction

Acute appendicitis is a common surgical emergency in the elderly. In the United States, the incidence of acute appendicitis in the 60-69-year cohort is only about half as frequent as its peak incidence in the 10-19-year age group [1]. Prostate cancer is the most common nonskin cancer in men [2]. Approximately 60% of cases of prostate cancer are diagnosed in men over 65 years [3]. Metastatic neoplasms are an important cause of acute appendicitis in older adults. The occurrence of acute appendicitis in a patient with an abdominopelvic malignancy, especially when treatment-resistant, should prompt consideration of secondary spread of the neoplasm to the appendix [4].

## 2. Case Presentation

The patient is a 66-year-old man who presented to the emergency department with a history of sudden onset of severe generalized abdominal pain of two-day duration associated with tenderness, fever (99°F), chills, nausea, and decreased appetite. The pain originated in the epigastric region, migrated to the right lower quadrant, and then became generalized. The pain was mostly constant but was also associated with intermittent episodes of slow, spontaneous improvement. The pain was worsened by sudden movement and oral intake. A known diabetic, home blood glucose measurements revealed concomitant increased levels despite his reduced oral intake.

His medical history is notable for a primary Gleason pattern 4 and secondary Gleason pattern 3 (Gleason Score of 7) prostatic adenocarcinoma diagnosed seven years earlier. His tumor had several adverse oncologic features, including advanced stage (stage pT3b), high tumor volume (involved 60% of the prostate), multifocal extraprostatic extension, small vessel invasion, positive surgical margins, and BRCA2 mutation positivity.

Following an initial radical prostatectomy, he received radiotherapy and then hormonal therapy with leuprolide for persistent increase (up to 2.28 *μ*g/L) in serum prostate-specific antigen (PSA). Leuprolide was discontinued following normalization of his PSA (to 0.0 *μ*g/L) but was recommenced two years later with the addition of Enzalutamide due to the recurrence of PSA elevation (7.79 *μ*g/L). The dose of Enzalutamide was subsequently reduced due to intolerable adverse effects. Bone and computed tomography (CT) scans showed no evidence of metastatic disease.

At presentation, physical examination was notable for abdominal distention and tenderness, without guarding or rebound tenderness. Complete blood count at admission showed a decreased white blood cell count of 9 × 10^9^ L^−1^ (reference range: 4.2 − 9.1 × 10^9^ L^−1^) with an increased neutrophil count of 7.3 × 10^9^ L^−1^ (reference range: 1.8 − 5.4 × 10^9^ L^−1^) and a decreased lymphocyte count of 0.9 × 10^9^ L^−1^ (reference range: 1.3 − 3.6 × 10^9^ L^−1^), anemia with hemoglobin of 117 g/L (reference range: 137-175 g/L), and elevated serum glucose of 7.82 mmol/L (reference range: 3.31-5.45 mmol/L). CT scan showed an enlarged, inflamed appendix with inflammatory changes in the mesoappendix.

**Figure 1:**
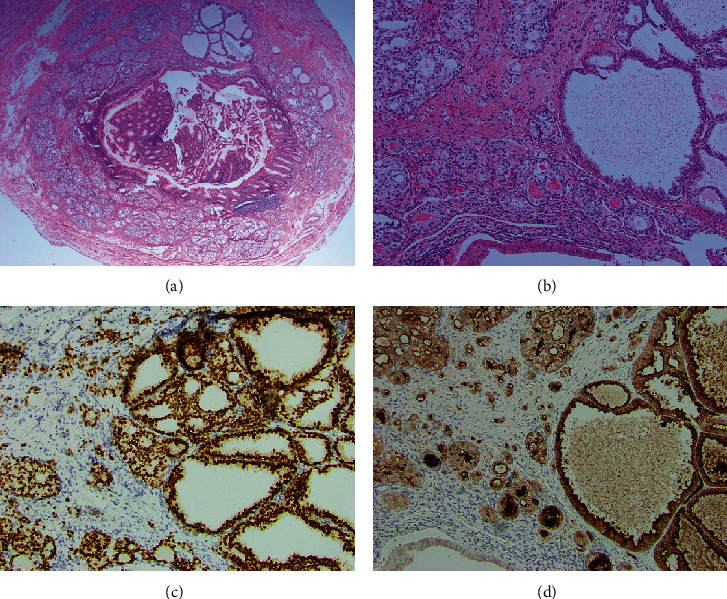
(a) A representative histologic section of the appendix. At scanning magnification, a gland-forming neoplasm composed of tightly-packed acini involves the appendix from the mucosa to the muscularis propria, H&E ×20. (b) At higher magnification, the tumor demonstrates variably sized, but predominantly small, back-to-back acini composed of cells with clear to amphophilic cytoplasm and enlarged nuclei. Amphophilic secretion is present in some tumor lumina, H&E ×100. (c) The tumor cells show strong and diffuse nuclear positivity for NKX3.1, confirming the diagnosis of metastatic prostatic adenocarcinoma, ×100. (d) The tumor cells also show strong cytoplasmic immunopositivity for prostate-specific antigen, further confirming the diagnosis. The luminal epithelium of the appendix (bottom left of the image) is unstained, providing a negative control for the test, ×100.

Following the administration of intravenous antibiotics, an appendectomy was performed without complications. Findings at surgery included an inflamed appendix and dense inflammatory adhesions between bowel loops and the mesentery. On gross pathologic examination, there were gray-green serosal and mesenteric exudates, but the appendix was not perforated. The lumen was dilated and contained clear mucus secretions. Histopathologic examination showed malignant epithelial cells arranged in crowded tubules extending from the lamina propria to the muscularis propria (Figures 1(a) and 1(b)), with involvement of the resection margins. Positive immunolabeling of the neoplasm for NKX3.1 (Figure 1(c)) and PSA (Figure 1(d)) confirmed the diagnosis of metastatic prostatic adenocarcinoma. The background appendix showed marked transmural acute inflammation and diverticula.

The patient's postoperative course has been notable for weakness, fatigue, and an unsteady gait. But he has a good appetite and no weight loss and continues working at this job.

## 3. Discussion

Bone is the most common site of metastasis from prostatic carcinoma [5]. Other common sites of distant spread of prostatic carcinoma include distant lymph nodes, liver, and thorax (including the lung, pleura, and mediastinum) [5]. The appendix is an unusual site of metastatic spread of prostatic adenocarcinoma. Other uncommon sites of spread of prostatic adenocarcinoma include the breast, brain, stomach, ureter, and rectum [6–8].

Acute appendicitis is the second most common cause of acute abdomen requiring surgical intervention in patients 50 years of age and older [9]. Malignancy is an important, albeit rare, cause of acute appendicitis in older adults [10, 11]. Studies have found neoplasms in up to 2.5% appendectomy specimens [12, 13], with only about 0.1% being metastatic [14, 15]. Neuroendocrine tumors are the most common primary appendiceal neoplasms associated with acute appendicitis [11, 12, 14]. Other primary appendiceal neoplasms identified in the causation of acute appendicitis include adenomas (sessile serrated adenoma, tubular adenoma, and villous adenoma), adenocarcinoma, and low-grade and high-grade appendiceal mucinous neoplasms [11, 12, 15].

Secondary involvement of the appendix by a malignant tumor may occur by either lymphohematogenous spread, peritoneal dissemination [16], or direct extension [17]. Metastases to the appendix arise most commonly from primary colorectal [14] and ovarian tumors [17] but can also originate from primary tumors in virtually any organ, including the breast [18], lung [19], stomach [16, 20], small bowel [21], pancreas [22], liver [23], endometrium [24], and the prostate gland [4]. Up to 97% of patients with appendiceal neoplasms have a preoperative diagnosis of acute appendicitis [13]. Luminal obstruction is the suggested underlying mechanism of appendiceal inflammation in metastatic disease [22]. The presence of a dilated, mucin-filled appendiceal lumen in the index case supports this position.

With only rare reports of secondary spread of prostatic carcinoma to the appendix, metastatic disease might not be uppermost in consideration in elderly patients with acute appendicitis. However, the simultaneous existence of a biochemically recurrent malignancy with multiple adverse oncologic factors presents the perfect backdrop for the manifestation of this rare phenomenon. An appendiceal metastasis should thus be strongly considered in such situations.

Suspicion for an underlying metastatic neoplasm will aid in the planning of appropriate treatment. For example, the administration of antibiotics is the sole treatment modality in selected patients with acute appendicitis [25], but this might not be the best option in the setting of metastatic disease. A consideration of neoplasia might also lead to a modification of the surgical approach, such as a more thorough examination of the bowel and peritoneum for suspicious lesions. Outcome after resection has been varied, with some studies showing no improvement in survival after complete resection of the secondary tumor [17], while others show good outcomes on short-term follow-up [16].

## References

[1] Buckius M. T., McGrath B., Monk J., Grim R., Bell T., Ahuja V. (2012). Changing epidemiology of acute appendicitis in the United States: study period 1993-2008. *The Journal of Surgical Research*.

[2] Siegel R. L., Miller K. D., Jemal A. (2018). Cancer statistics, 2019. *CA: a Cancer Journal for Clinicians*.

[3] Rawla P. (2019). Epidemiology of prostate cancer. *World Journal of Oncology*.

[4] Khan K., Rodriguez R., Landa M., Davis-Joseph B. (2018). Appendicitis: a rare case caused by metastatic prostate cancer. *Urology Case Reports*.

[5] Gandaglia G., Abdollah F., Schiffmann J. (2014). Distribution of metastatic sites in patients with prostate cancer: a population-based analysis. *Prostate*.

[6] Mandaliya H., Sung J., Hill J., Samali R., George M. (2015). Prostate cancer: cases of rare presentation and rare metastasis. *Case Reports in Oncology*.

[7] Soe A. M., Bordia S., Xiao P. Q. (2014). A rare presentation of metastasis of prostate adenocarcinoma to the stomach and rectum. *Journal of Gastric Cancer*.

[8] Chung H. S., Kim M. S., Cho Y. H. (2017). A rare presentation of metastatic prostate cancer, initially a suspect for urothelial cell carcinoma of the ureter: a case report. *BMC Urology*.

[9] Kraemer M., Franke C., Ohmann C., Yang Q. (2000). Acute appendicitis in late adulthood: incidence, presentation, and outcome. Results of a prospective multicenter acute abdominal pain study and a review of the literature. *Langenbeck's Archives of Surgery*.

[10] Shine R. J., Zarifeh A., Frampton C., Rossaak J. (2017). Appendicitis presenting as the first manifestation of colorectal carcinoma: a 13-year retrospective study. *NZMJ*.

[11] Brunner M., Lapins P., Langheinrich M. (2020). Risk factors for appendiceal neoplasm and malignancy among patients with acute appendicitis. *International Journal of Colorectal Disease*.

[12] Kunduz E., Bektasoglu H. K., Unver N., Aydogan C., Timocin G., Destek S. (2018). Analysis of appendiceal neoplasms on 3544 appendectomy specimens for acute appendicitis: retrospective cohort study of a single institution. *Medical Science Monitor*.

[13] Esmer-Sanchez D. D., Martinez-Ordaz J. L., Roman-Zepeda P., Sanchez-Fernandez P., Medina-Gonzalez E. (2004). Appendiceal tumors. Clinicopathologic review of 5,307 appendectomies. *Cirugia y Cirujanos*.

[14] Connor S. J., Hanna G. B., Frizelle F. A. (1998). Appendiceal tumors: retrospective clinicopathologic analysis of appendiceal tumors from 7,970 appendectomies. *Diseases of the Colon and Rectum*.

[15] Kinnear N., Heijkoop B., Bramwell E. (2019). Communication and management of incidental pathology in 1,214 consecutive appendicectomies; a cohort study. *International journal of surgery*.

[16] Simpson G. S., Mahapatra S. R., Evans J. (2013). Incidental complete excision of appendiceal gastric cancer metastasis. *Journal of Surgical Case Reports*.

[17] Yoon W. J., Yoon Y. B., Kim Y. J., Ryu J. K., Kim Y. T. (2010). Secondary appendiceal tumors: a review of 139 cases. *Gut and Liver*.

[18] Araujo J., Cavalcanti B., Soares M., Sousa U., Medeiros G. (2018). Metastases of breast cancer causing acute appendicitis: a case report. *Cancer Reports and Reviews*.

[19] Wolf C., Friedl P., Obrist P., Ensinger C., Gritsch W. (1999). Metastasis to the appendix: sonographic appearance and review of the literature. *Journal of Ultrasound in Medicine*.

[20] Karanikas M., Kofina K., Markou M. (2018). Acute appendicitis as the first presentation of appendiceal metastasis of gastric cancer-report of a rare case. *Journal of Surgical Case Reports*.

[21] Bandyopadhyay D., Bonatti H. J. R. (2019). Acute right lower abdomen in a patient with a history of gastrointestinal stromal tumor. *Case Reports in Surgery*.

[22] Filik L., Ozdal-Kuran S., Cicek B., Zengin N., Ozyilkan O., Sahin B. (2003). Appendicular metastasis from pancreatic adenocarcinoma. *International Journal of Gastrointestinal Cancer*.

[23] Kim H. C., Yang D. M., Jin W., Kim G. Y., Choi S. I. (2008). Metastasis to the appendix from a hepatocellular carcinoma manifesting as acute appendicitis: Ct findings. *The British Journal of Radiology*.

[24] Ma Q., Wu J. (2019). Endometrioid adenocarcinoma with solitary metastasis to the appendix, mimicking primary appendiceal adenocarcinoma: a case report and literature review. *International Journal of Gynecological Pathology*.

[25] Talan D. A., Saltzman D. J., DeUgarte D. A., Moran G. J. (2019). Methods of conservative antibiotic treatment of acute uncomplicated appendicitis: a systematic review. *Journal of Trauma and Acute Care Surgery*.

